# Frequency of Hemorrhagic Side Effects of Botulinum Neurotoxin Treatment in Patients with Blepharospasm and Hemifacial Spasm on Antithrombotic Medication

**DOI:** 10.3390/toxins14110769

**Published:** 2022-11-07

**Authors:** Fiona Carolin Wenninger, Bettina Wabbels

**Affiliations:** 1 Department of Ophthalmology, University Hospital of Bonn, Ernst-Abbe-Str. 2, D-53127 Bonn, Germany; 2 Department of Neurology, University Hospital of Münster, Albert-Schweitzer-Campus 1, D-48149 Münster, Germany

**Keywords:** hematoma, botulinum neurotoxin, blepharospasm, hemifacial spasm, antithrombotics, anticoagulants

## Abstract

The aim of this study was to investigate the frequency of hemorrhagic side effects of botulinum neurotoxin A injections (BoNT/A) for the treatment of benign essential blepharospasm (BEB) and hemifacial spasm (HFS) in patients taking antithrombotic drugs (ATD). A total of 140 patients were included (female: 65%; BEB: 75%; mean age: 70 ± 12 years). According to their current antithrombotic medication, participants were either assigned to the ATD group (41%), or to the control group (59%). The ATD group was further divided into subgroups depending on the medication administered: acetylsalicylic acid, ADP receptor antagonists, direct oral anticoagulants, vitamin-K antagonists, or dual antiplatelet therapy. The frequency of hemorrhagic side effects was recorded by retrospective analysis of past treatments as documented in the patient’s file set in relation to the number of past treatments (hematoma frequency of past treatments, HF_retro_) as well as by a prospective survey capturing the side effects of one single treatment (hematoma frequency of actual treatment, HF_actual_). There was no significant difference in hematoma frequency between the ATD group and the control group, neither for past (HF_retro_: ATD: 2%; 45/2554; control: 4%; 109/2744) nor for the current BoNT/A treatments (HF_actual_: ATD: 30%; 16/53; control: 31%; 22/72). Even between ATD subgroups, hematoma frequency did not differ significantly. Overall, hemorrhagic side effects of the BoNT/A treatment for BEB and HFS were mild and non-disabling.

## 1. Introduction

Benign essential blepharospasm (BEB), a focal dystonia, is characterized by increased blinking due to overactivity of the orbicularis oculi muscle. In the course of time the involuntary muscle contractions may be prolonged, causing functional blindness [1,2,3,4], and may spread to neighboring muscles (Meiges syndrome) [5,6]. Up until now the underlying cause of the disease remains unknown. Depending on the geographical region, the prevalence of BEB varies between 1.7 and 13.3 per 100,000 [7,8]. Women are predominantly found among those affected. The first symptoms usually appear between the fifth and seventh decade of life [9,10].

Hemifacial spasm (HFS) is characterized by mostly unilateral, involuntary contractions of the facial muscles innervated by the facial nerve. These can appear as slight muscle tremors, significant twitching or long-lasting muscle spasms of individual facial muscle groups or half of the face [11,12,13,14]. HFS usually occurs due to compression of the facial nerve [15] caused by neighboring arterial vessels of the brainstem and cerebellum as well as masses, cysts, or bony abnormalities [16,17,18]. According to Rosenstengel et al. (2012), approximately 8000–9000 people in Germany suffer from HFS [19], corresponding to a prevalence of 9.9–11.8 per 100,000 inhabitants. Women are particularly affected [19,20], as are people over the age of 40 years [20,21].

The unsatisfactory effects of numerous oral drugs [13,22] and a significantly higher complication rate of surgical interventions [1,13] make regular injections of BoNT/A the therapy of first choice for BEB and HSF [22,23]. For this purpose, three different preparations of BoNT/A are currently available in Germany: onabotulinum toxin (Botox^®^, Allergan Pharmaceuticals, Dublin, Ireland), incobotulinum toxin (Xeomin^®^, Merz Pharmaceuticals GmbH, Frankfurt/Main, Germany) and abobotulinum toxin (Dysport^®^, Ipsen Pharma, Paris, France). To treat BEB and HFS, a combined subcutaneous-intramuscular injection of the toxin in affected muscles is carried out. Since the paralytic effect is only temporary, the treatment must be repeated at regular intervals of usually eight to twelve weeks [24].

Compared to the high therapeutic effect, adverse effects of BoNT/A treatment occur rarely, are mostly mild, and are only of short duration [13,25]. This also applies to subcutaneous bleeding and hematomas around the injection sites. However, there is concern that patients on antithrombotic medication are at higher risk of subcutaneous bleeding and hematoma. Antithrombotic therapy is used to prevent or treat thrombosis in patients in a variety of clinical situations of different cardiovascular conditions [26], and includes two major classes of drugs: anticoagulants and platelet aggregation inhibitors. Antiplatelet agents prevent or delay the activation and aggregation of platelets and include acetylsalicylic acid (ASA) and ADP receptor antagonists (ADP-RA) [27,28], while a combination of platelet aggregation inhibitor drug groups is referred to as dual antiplatelet therapy (DAPT). Anticoagulants prevent or delay the formation of a red thrombus through activated coagulation factors and include vitamin K antagonists (VKA) and direct oral anticoagulants (DOAC).

According to the manufacturers of vitamin K antagonists (VKA), the subcutaneous-intramuscular injection of BoNT/A in patients undergoing therapy with these preparations is contraindicated “due to the risk of massive bleeding” (direction for use Marcumar^®^ 3 mg tablets, MEDA Pharma GmbH & Co. KG, Bad Homburg, Germany, 2018; direction for use Phenpro.-ratiopharm^®^ 3 mg tablets, ratiopharm GmbH, Ulm, Germany 2018). The respective directions for use of other antithrombotics do not include this contraindication explicitly but include a warning about hematoma. However, due to lifestyle and demographic changes with an aging population, the number of patients with antithrombotic medication is expected to increase. At the same time, the number of studies investigating the risk of bleeding after BoNT/A injection in patients on antithrombotic medication is limited to date. The aim of this study was to assess the frequency and severity of hemorrhagic side effects of BoNT/A treatment for BEB and HFS in patients on antithrombotic medication. Special attention was paid to the medical relevance of these adverse effects and their influence on the physical and psychological well-being of the affected patients.

## 2. Results

### 2.1. Demographic Data

A total of 140 patients were included in the study between May and November 2019. Overall, the mean (±SD) age of the participants was 70 ± 12 [37;91] years, with 65% (*n* = 91) being women. Most participants underwent BoNT/A treatment for BEB (75%, 105/140). In median, participants had already been treated at the University Eye Clinic Bonn for 9 [0;33] years. The number of past BoNT/A treatments, including the current treatment, varied between two and 155 treatments, with a median of 35 treatments per study participant. For the current treatment, 70% (73/105) of the participants suffering from BEB were treated with Xeomin*^®^*, whereas Botox*^®^* was mostly used to treat HFS (69%, 24/35). The median total dose of the BoNT/A preparation administered for the treatment of BEB was 35.0 [7.5;75.0] units of Botox*^®^* or 30.0 [5.0;85.0] units of Xeomin*^®^*. A median of 12.5 [7.5;30.0] units of Botox*^®^* and 20.0 [7.5;65.0] units of Xeomin*^®^* were administered for the treatment of HFS. Antithrombotic medication was taken by 41% (58/140) of the participants.

Table 1 shows demographic and treatment characteristics of patients in the control group and the ATD group. Apart from age (*p* < 0.01) and the applied BoNT/A preparation (*p* = 0.04), there were no significant differences between groups. The most frequently used antithrombotic drug was ASA (52%, 30/58). Almost every fourth participant on antithrombotic medication claimed to be on DOAC (24%, 14/58). The regular intake of VKA, ADP-RA or DAPT was much less common. Table 2 shows demographic and treatment characteristics of patients in the ATD group subgroups. Except for gender distribution (*p* = 0.01), there was no significant difference between the subgroups of the ATD group.

### 2.2. Hematoma Frequency

More than one-third of patients had at least once experienced a hematoma after treatment with BoNT/A in the past (HF_retro_) in both the control and the ATD group (control: 39%, 31/80; ATD: 38%, 22/58). Adjusting for the number of past BoNT/A treatments, HF_retro_ averaged 3% (154/5298, [0.0;100]) of treatments, with a mean of 4% (109/2744), [0.0;100] within the control group and 2% (45/2554, [0.0:33.3] in the ATD group (Table 3). The files of two participants in the control group were incomplete regarding the history of side effects and were not included in the analysis.

Overall, the proportion of patients with hematoma after current BoNT/A treatment (HF_actual_) was 30.4% (38/125). Participants in both the control and ATD group were equally affected by hematoma after current BoNT/A treatment (HF_actual_: control: 31%, 22/72; ATD: 30%, 16/53). Within the subgroups ADP-RA and DAPT, half of all participants suffered a hematoma after current BoNT/A injection (ADP-RA: 50%, 2/4; DAPT: 50%, 1/2), whereas for the participants who regularly took a DOAC the HF_actual_ was 8% (1/13). Despite the evidence of these tendencies, a significant connection between the intake of certain antithrombotic drugs and the occurrence of a hematoma after current treatment could not be demonstrated (Table 3). A total of 15 participants (control: 10; ATD: 5) did not complete the questionnaire on current BoNT/A treatment and were not included in the analysis.

### 2.3. Hematoma Intensity

The majority of participants (42%, 15/36) reported that the hematoma occurred immediately after treatment or on the same day (47%, 17/36). Only 11% (4/36) of the patients with hematoma, all of whom belonged to the ATD group, stated that the hematoma appeared one or more days after the BoNT/A injection. The hematomas were visible for a mean period (±SD) of 9.9 ± 7.4 [3.0;33.0] days. There was no significant difference between the control and ATD group or between the ATD subgroups regarding the duration of the hematoma. Two participants in the control group did not provide information on the duration of the hematoma and were therefore not included in this analysis.

**Figure 1:**
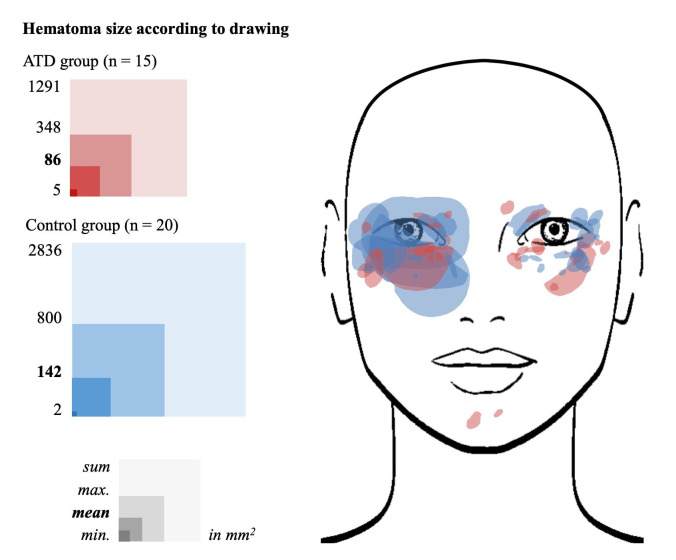
Depiction of hematomas drawn in a facial image after current BoNT/A injection according to their size and localization. Overview of mean, maximum and minimum of total hematoma area per participant and sum of total hematoma area per group in comparison of the control and ATD groups.

More than half of the participants stated that the hematoma was punctiform and ≤1.5 cm in diameter (57%, 21/37). The other participants reported more pronounced hematomas, however mostly ≤2.5 cm in diameter (24%, 9/37). The size of the hematoma drawn in a facial image varied between two and 800 mm^2^ per participant, with a mean (±SD) of 118 ± 203 mm^2^. There was no significant difference between the control and ATD group or between the ATD subgroups regarding the size of the hematoma based on drawings (Figure 1). One participant in the control group did not provide information on the size of the hematoma and was therefore not included in this analysis.

Overall, 58 individual hematomas were drawn by 35 participants. Accordingly, a mean of 1.6 ± 0.8 [1;4] hematomas occurred per affected participant. The number of hematomas following current BoNT/A injection differed significantly (*p* = 0.02) between control and ATD groups. Participants in the control group reported a mean of 1.4 ± 0.7 [1;3] individual hematomas, while participants in the ATD group suffered from a mean of 2.0 ±0.9 [1;4] hematomas. However, no significant difference in the number of hematomas after current BoNT/A treatment was observed between ATD subgroups.

### 2.4. Hematoma Consequence

On a visual analogue scale (VAS) the mean (±SD) impairment caused by the hematoma was reported as 1.4 ± 2.2 [0.0;7.6], with most of the participants (49%, 16/33) feeling completely unaffected (VAS: 0). There was no significant difference between the ATD group (VAS: 1.4 ± 2.3 [0.0;7.0]) and the control group (VAS: 1.4 ± 2.2 [0;7.6.0]) or between ATD subgroups. Cosmetic reasons (21%, 7/33), pain, or a feeling of pressure (9%, 3/33) were given as reasons for the impairment caused by the hematoma. To deal with the disturbing side effects, 27% (10/37) of those affected took further measures such as cooling affected areas (22%, 8/37). Five participants (control: 4; ATD: 1) did not provide information about impairment and were therefore not included in this analysis.

### 2.5. Non-Hemorrhaghic Side Effects

Regarding past BoNT/A treatments in 84% (118/138) of patients records other side effects than hematoma were noted. The most common were ptosis (34%, 47/139), lacrimation (33%, 46/139), and unspecified visual disturbances (27%, 38/139). For two patients, this information was not available.

Regarding the current BoNT/A treatment, most participants (60%, 74/124) reported no side effects apart from hematoma. The most common included tearing (6%, 7/124) and burning, pain, and dry eye sensation (4% each, 5/124).

## 3. Discussion

Our study shows that BoNT/A injections for the treatment of BEB and HFS are not associated with more frequent hemorrhagic side effects in patients on ATD, regardless of the agent, than in patients without—both in the retrospective analysis of past BoNT/A treatments and in the prospective survey on current BoNT/A injections. Except the number of single hematomas per patient, there is also no significant difference in the severity of hematomas occurring after BoNT/A treatment between patients on ATD, regardless of the agent, and patients without.

However, the retrospectively determined hematoma frequency of past BoNT/A treatments (HF_retro_) was significantly lower than of the current BoNT/A injection (HF_actual_)—both in the overall cohort of participants (*p* < 0.01) and within the control (*p* < 0.01) and ATD group (*p* < 0.01).

Regarding past BoNT/A treatments (HF_retro_), overall, a hematoma frequency of 3% was determined. Various studies on the effectiveness and safety of different BoNT/A preparations for the treatment of BEB and HFS revealed comparable results (Table 4). Bentivoglio et al. (2009) retrospectively determined a hematoma frequency of 3.2% (43/1341) after treatment of BEB [29]. Jankovic and colleagues (2011) also observed a comparable hematoma frequency in patients with HFS [30]. In contrast, the hematoma frequency of 25.0% (16/64), determined by Wabbels et al. (2010) was considerably higher and corresponds to HFS_actual_ (30%) in our study [31].

The heterogeneous data on frequency of hematoma after BoNT/A treatment within our study and within the literature (Table 4) could be explained by different study designs. Presumably, small, only briefly visible and non-burdening hematomas are often forgotten and therefore only remembered when asked explicitly and in time, as in the case of determination of HF_actual_. In contrast to this, determination of the HF_retro_ as well as the hematoma frequency in numerous other studies was carried out by an open-ended questioning about side effects of past BoNT/A treatments without particular attention to hemorrhagic complications. In addition, the data collection usually took place several weeks after the treatment in question. It can therefore be assumed that, both in determination of the HF_retro_ in our study and the hematoma frequency in comparable studies that followed a retrospective study design, fewer hematomas were recorded than actually occurred.

Given the large difference between HF_actual_ and Hf_retro,_ we consider it unlikely that the higher hematoma frequency of current BoNT/A treatments was randomly generated by case number differences (current treatment: *n* = 125; past treatments: *n* = 5298). Since participants received the current BoNT/A injection by different physicians, a physician-related increased hematoma frequency can also be ruled out.

To date, very few studies investigated the hematoma frequency after BoNT/A treatment in patients on antithrombiotic medication. Schrader and colleagues (2018) retrospectively determined the hematoma frequency of BoNT/A treatment for BEB, HFS, cervical dystonia and stroke-related spasticity in patients on phenprocoumon. After a total of 231 and 206 BoNT/A treatments for BEB and HFS, respectively, there was no significant difference in hematoma frequency between patients on phenprocoumon and their matched controls (BEB: 5.2% vs. 2.6%; HFS: 3.9% vs. 2.9%). These results largely correspond to the HF_retro_ of the control and VKA group in our study. In contrast, the HF_actual_ of both groups in our study is considerably higher. The comparison of these results confirms the already discussed assumption that an open-ended and delayed questioning of patients, as performed when determining the hematoma frequency in the study of Schrader et al. and the HF_retro_ in our study, underestimates the real hematoma frequency.

Furthermore, Jagatsinh and George (2012) investigated the safety of different BoNT/A preparations for the treatment of spastic disorders on warfarin [41]. After a total of 103 intramuscular injections, none of the 14 participants registered hemorrhagic complications. The hematoma frequency to be calculated would therefore be 0%, and corresponds to the HF_retro_ of the VKA group in our study. Again, the HF_actual_ of the VKA group determined in our study is considerably higher than the hematoma frequency determined by Jagatsinh and George. However, in view of the small number of participants on phenprocoumon in our study, the comparison of the results is only possible to a very limited extent. In addition, the comparability of the study results might be limited by the different pharmacological properties of the VKA warfarin and phenprocoumon [24,42,43,44].

There are no studies on the frequency of hematomas after BoNT/A treatment on other antithrombotics such as DOAC, ADP-RA or ASA. However, according to the direction for use for the preparations in question, hematomas or bleeding generally after medical interventions and injections or punctures occur “rarely” to “frequently” (≥1/10,000 to <1/10) depending on the respective preparation. This corresponds to a hematoma frequency between ≥0.01% and <10.0%, and is therefore comparable to the HF_retro_ determined in our study to be between 0% (ADP-RA) and 2% (ASA).

## 4. Conclusions

In view of the results of our study, pausing antithrombotic medication with ASA, VKA or DOAC in the context of BoNT/A treatment for BEB and HFS does not seem justified. However, since only a small number of patients on ADP-RA and DAPT were included in our study, subsequent studies are necessary to be able to make a recommendation for these agents. For future studies, it should be considered that timing and questioning technique may lead to significant differences in the reported frequency and description of hemorrhagic side effects.

Overall, our study showed that hemorrhagic side effects of the BoNT/A treatment for BEB and HFS are mild and non-disabling.

## 5. Materials and Methods

The study was approved by the local ethics committee of the “Rheinische Friedrichs-Wilhelms-Universität Bonn” and has been performed in accordance with the ethical standards of the Declaration of Helsinki and its later amendments. All participants provided a signed informed consent form for participation in this study. Participation did not affect the patient’s medical care. Antithrombotic treatment was continued as prescribed and therefore was not influenced by the study.

Patients with BEB or HFS undergoing regular treatment with BoNT/A at the University Eye Clinic in Bonn were consecutively included in the study. According to their medication, the participants were assigned to the following study groups: the control group (no intake of antithrombotic drugs), the ATD group (regular intake of antithrombotic drugs), and their subgroups regarding the antithrombotic agent (ASA, ADP-RA, DAPT, DOAC or VKA).

The frequency of hematomas caused by BoNT/A treatment for BEB and HFS was recorded for two separate periods: in a retrospective analysis of past BoNT/A treatments (HF_retro_), and in a prospective survey on one single BoNT/A treatment (HF_actual_).

The HF_retro_ was calculated for each participant and each study group from the absolute number of past BoNT/A treatments that caused a hematoma in relation to the total number of BoNT/A treatments in our clinic. Information on adverse events including hematoma following past BoNT/A treatments was collected from patient records. Adverse events are queried in a standardized manner at every follow-up visit in our clinic (about 8–12 weeks after the respective treatment) and documented in the patient file. The patient records were also used to collect data on demographics, diagnosis (BEB or HFS), specifics of the BoNT/A therapy performed in our eye clinic (duration, number of injections administered, type of BoNT/A), comorbidities, and medication.

The HF_actual_ was calculated for each study group from the number of participants who suffered a hematoma after a single, currently performed BoNT/A treatment in relation to the total number of participants in the group. The mentioned BoNT/A treatment was performed by different physicians according to the patient’s current treatment regimen. In order to ensure that the injection was carried out as usual and without special care, the attending physician was not informed about the patient’s participation in the study. Post-treatment, participants were given a questionnaire to assess the occurrence, size, location, onset, and duration of hematoma, as well as the occurrence of non-hemorrhagic adverse events after this single BoNT/A injection. To assess the hematoma size in an objective way, participants were asked to compare the hematoma to a 1 cent coin (about 1.5 cm in diameter, 207 mm^2^), a 2 euro coin (about 2.5 cm in diameter, 520 mm^2^) or a quarter of the face. In addition, the area of hematomas drawn in the facial image was calculated by creating representative polygons using the SketchAndClac software (Dobbs, Elliott M. “www.SketchAndCalc.com” (accessed on 12th November 2021). SketchAndCalc. Elliott M Dobbs, 20 February 2011. Web, version 4.1.8.11.). A visual analogue scale (VAS) ranging from 0 (no impairment) to 10 (worst impairment) was used to assess how much the hematoma bothered the patients. Patients were asked to complete the questionnaire within two weeks of the treatment in question and to return it to our clinic.

Statistical analysis of the pseudonymized data was performed using the statistical program IBM SPSS Statistics, version 26 (IBM Corp., Armonk, NY, USA). Results were expressed as mean, standard deviation, minimum, median, quartiles, and maximum. For determining statistical significance between groups, the Mann-Whitney-Test (control vs. ATD group) and the Kruskall-Wallis-Test (between ATD subgroups) were performed (*p* < 0.05 considered statistically significant).

## Figures and Tables

**Table 1:** Comparison of demographic and therapy-related characteristics of the participants in the control and ATD group.

Characteristics		Control Group (*n* = 82)	ATD Group (*n* = 58)
Number of participants	n (%)	82 (59)	58 (41)
Age (years)	mean ± SD	66 ± 11	74 ± 10
Female	n (%)	58 (71)	33 (57)
BEB	n (%)	60 (73)	45 (78)
Xeomin^®^	n (%)	55 (67)	29 (50)
Number of treatments	median [R]	29 [2;155]	41 [2;137]

ATD, antithrombotic drug; BEB, benign essential blepharospasm.

**Table 2:** Comparison of demographic and therapy-related characteristics of participants taking antithrombotic medication according to subgroup.

Characteristics		ASA	DOAC	VKA	ADP-RA	DAPT
Number of participants	n (%)	30 (52)	14 (24)	8 (14)	4 (7)	2 (4)
Age (years)	mean ± SD	73 ± 10	77 ± 9	73 ± 16	82 ± 5	73 ± 5
Female	n(%)	14 (47)	12 (59)	3 (38)	4 (100)	0 (0)
BEB	n(%)	22 (73)	11 (79)	7 (88)	3 (75)	2 (100)
Xeomin^®^	n(%)	16 (67)	6 (43)	4 (40)	2 (50)	1 (50)
Number of treatments	median [R]	41 [2;107]	46 [2;120]	29 [2;107]	25 [17;77]	6 [4;8]
Treatment duration (years)	median [R]	12 [0;33]	14 [0;24]	13 [2;32]	5 [4;26]	2 [2;2]

ASA, acetylsalicylic acid; ADP-RA, adenosine diphosphate receptor antagonists; DAPT, dual antiplatelet therapy; DOAC, direct oral anticoagulants; VKA, vitamin K antagonists.

**Table 3:** HF_retro_ and HF_actual_ based on the number BoNT/A treatments (T) and the number of following hematoma events (H) in control and ATD group as well as ADT subgroups.

Group		Past Treatments				Current Treatment		
		T	H	HF_retro_		T	H	HF_actual_
	n (%)	n	n	Mean %	[R %]	n (%)	n	(%)
**Control**	80 (58) ^a^	2744	109	4	[0.0;100]	72 (58) ^b^	22	31
**ATD**	58 (42)	2554	45	2	[0.0;33.3]	53 (42)	16	30
ASA	30 (52)	1231	24	2	[0.0;6.0]	27 (51) ^b^	11	41
DOAC	14 (24)	730	7	1	[0.0;6.0]	13 (25) ^b^	1	8
VKA	8 (14)	443	13	0	[0.0;14.5]	7 (13) ^b^	1	14
ADP-RA	4 (7)	140	0	0	[0.0;0.0]	4 (8)	2	50
DAPT	2 (7)	10	1	0	[0.0;33.3]	2 (4)	1	50
**total**	138 (100) ^a^	5298	154	3	[0.0;100]	125 (100)	38	30

^a^ Due to missing information, two participants of the control group were not included in the determination of the absolute number of past hematoma events and HF_retro_. ^b^ 10 participants of the control group, three participants on ASA, and one each on DOAC or VKA did not return the questionnaire and were therefore not included in the determination HF_actual_. ASA, acetylsalicylic acid; ADP-RA, adenosine diphosphate receptor antagonists; DAPT, dual antiplatelet therapy; DOAC, direct oral anticoagulants; VKA, vitamin K antagonists.

**Table 4:** Overview of the hematoma frequency (HF) determined in past studies from the number of observed treatments (T) and subsequently among the patients (P) In descending order.

Subject of Study	P [n]	T [n]	H [n]	HF [%]	Methods ^a^	Reference
Hematoma frequency after current treatment of BEB and HFS (our study)	125	125	38	30	questionnaire ≤14 days explicit	(HF_actual_)
Hematoma frequency after past treatments of BEB and HFS (our study)	138	5298	154	3	patient’s record following session open	(HF_retro_)
Efficacy and safety of BOTOX^®^ versus Xeomin^®^ for the treatment of BEB	64	64	16	25,0	Interview ≥4 weeks open	Wabbels et al., 2010 [31]
Comparison of the effectiveness of preseptal and pretarsal injections of BOTOX^®^ for the treatment of BEB and HFS	40	80	4	5,0	Report ≥1 month open	Lolekha et al., 2017 [32]
Hematoma frequency of BoNT/A treatment of BEB or HFS taking phenprocoumon versus control group	28	437	19	4,4	patient’s record open	Schrader et al., 2018 [33]
Clinic features and treatment options of BEB including treatment with BoNT/A	151	-	≥ 5 ^b^	≥3,3 ^c^	patient’s record open	Grandas et al., 1988 [1]
Efficacy and safety of BOTOX^®^ versus Dysport^®^ in the treatment of BEB	-	1341	43	3,2	patient’s record open	Bentivoglio et al., 2009 [29]
Efficacy and safety of long-term BoNT/A treatment of BEB	234	10,632	340	3,1	patient’s record following session open	Wabbels et al., 2022 [34]
Efficacy and safety of treating HFS with Xeomin^®^ versus placebo	108	108	3	2,8	-	Jankovic et al., 2011 [30]
Efficacy and safety of BoNT/A treatment of BEB and HFS	83	241	6	2,5	report open	Jankovic et al., 1990 [35]
Efficacy and safety of BoNT/A treatment of BEB and HFS	112	212	≥2 ^b^	≥1,8 ^c^	report ≥3–6 days open	Park et al., 1993 [36]
Efficacy and safety of BoNT/A treatment of BEB and HFS	106	1028	10	1,0	questionnaire following session open	Hsiung et al., 2002 [37]
Efficacy and safety of BoNT/A treatment of BEB and HFS	131	920	≥7 ^b^	≥0,8 ^c^	patient’s record open	Cillino et al., 2010 [38]
Efficacy and safety of BoNT/A treatment of BEB and HFS	32	1421	≥2 ^b^	≥0,1 ^c^	patient’s record following session open	Ababneh et al., 2014 [39]
Efficacy and safety of treating HFS with BoNT/A versus placebo	288	10,701	0	0,0	patient’s record open	Kollewe et al., 2015 [40]

^a^ Data source, time of data collection after BoNT/A treatment and questioning technique used (open/explicit). ^b^ Number of participants with at least one hematoma after BoNT/A treatment. ^c^ Missing information on the number of hematoma events, therefore it is not possible to determine the exact hematoma frequency. BEB, benign essential blepharospasm; BoNT/A, botulinumtoxin A; HFS, hemifacial spasm.

## Data Availability

Data and materials can be provided by the authors upon reasonable request. The original data is included in paper files.
